# Managing retreat for sandy beach areas under sea level rise

**DOI:** 10.1038/s41598-023-38939-4

**Published:** 2023-07-24

**Authors:** Renee O. Setter, Rachael X. Han, Kammie-Dominique Tavares, Conrad Newfield, Alice Terry, Isabella M. Roberson, Nori Tarui, Makena Coffman

**Affiliations:** 1 Institute for Sustainability and Resilience, University of Hawai‘i at Mānoa, Honolulu, HI 96822 USA; 2 Department of Geography and Environment, University of Hawai‘i at Mānoa, Honolulu, HI 96822 USA; 3 Department of Urban and Regional Planning, University of Hawai‘i at Mānoa, Honolulu, HI 96822 USA; 4 University of Hawai‘i Economic Research Organization, University of Hawai‘i at Mānoa, Honolulu, HI 96822 USA; 5 Department of Economics, University of Hawai‘i at Mānoa, Honolulu, HI 96822 USA

**Keywords:** Climate-change adaptation, Governance, Climate-change policy

## Abstract

Sea level rise (SLR) is projected to impact approximately one billion people by 2100. For many coastal communities, retreat is the most viable long-term option due to exposure risk under SLR and increased coastal hazards. Our research analyzes the costs of retreating coastal development at an iconic beach in Hawaiʻi that is experiencing severe erosion. We assess three retreat approaches: all-at-once, threshold-based, and reactive. Utilizing detailed SLR modeling projected to the year 2100, we estimate the public and private costs of retreat approaches and the amount of increased beach area. We find an all-at-once approach is most costly but maintains the largest beach area over time. In contrast, a reactive approach has the lowest direct costs but offers the least beach area gained over time and incurs the greatest public safety and environmental risk. The threshold-based approach largely mitigates public safety and environmental risks while providing more beach area over time than the reactive approach with similar direct costs. We find that a threshold-based approach should be further explored as a SLR response for coastal communities to maintain their sandy beach areas. Our study informs coastal adaptation research and identifies a new framework to explore the financial costs alongside social and ecological values.

## Introduction

Global mean sea level is expected to rise by up to 1.01 m by 2100 due to a combination of thermal expansion of water and melting of glaciers and ice sheets^1–3^. Sea level rise (SLR) exacerbates the effects of other hazards, such as heavy rainfall and subsequent flooding, storm surge and high wave run-up, and coastal erosion, forcing coastal communities to adapt at an unprecedented rate^4,5^. Forty percent of the world’s population lives within 100 km of the coastline and approximately one billion people residing in coastal communities across the world are projected to be impacted by SLR by 2100, with assets valued at US$8–14 trillion ($2011)^6–8^. In addition to the direct impacts to people, infrastructure, and coastal ecosystems, there will be threats to food security as well as increasing disparities in social equity^9^.

There are three generic categories of SLR adaptation response: protect, accommodate, and retreat^10,11^. Protection is the defense and reinforcement of the shoreline to maintain existing land area^12,13^. Accommodation is a response where coastal risks are mitigated and coastal zones adapt by, for example, raising buildings^14^. Retreat is the relocation of buildings and people out of hazardous coastal areas^15,16^. While retreat can increase the long-term resilience of coastal communities, it is understudied and not applied as much as protection and accommodation^17^. Most SLR responses without policy intervention would result in in situ adaptation because of the prioritization of short-term economic benefits, maintenance of status quo, and a lack of public support for retreat^9,18^. However, there is a growing consensus that retreat will become an increasingly important strategy to adapt to SLR in many locations^15,18,19^.

Despite the urgent need for action, careful SLR response is necessary to avoid maladaptive outcomes^9,20^. The hardening of coastal areas with eroding sandy beachfronts is often viewed as maladaptation due to its exacerbation of coastal erosion, resulting in loss of the sandy beach and perpetuating increasing risk to the public^9^. Few studies quantify the costs of various SLR adaptation approaches, but rather broadly estimate the loss of assets to SLR and costs of protection^21–24^. This study is novel as we evaluate the costs of both managed and unmanaged coastal retreat approaches in residential areas adjacent to sandy beaches. We apply this to a case study within the Sunset Beach, Paumalū region on the North Shore of O‘ahu, Hawai‘i. We identify types of costs related to three different approaches to retreat over an eighty-year period through 2100, in comparison with relative potential beach area recovered under each approach.

## Study area

The coastal communities and ecosystems of Hawaiʻi are tremendously at risk from SLR and its compounding impacts such as coastal erosion, direct marine flooding, and groundwater inundation^25–29^. It is estimated that 1 m of SLR exposure will cover an area of 105 km^2^ of land in Hawaiʻi, which has a land and dwelling value of $19 billion ($2013)^30,31^. By the end of the century, half of Hawai‘i’s sandy shorelines may be at risk of beach loss, threatening native ecosystems, cultural practices, and recreational use^32–35^. SLR is also projected to threaten many homes and jeopardize major infrastructure such as wastewater systems and coastal highways, posing an increased public safety and health risk and disrupting community livelihoods^29,32,36–38^. Although there is little agreement on how to accomplish it, the preservation of the diverse uses and values of Hawaiʻi’s beaches in the face of SLR has been emphasized by coastal managers^39^.

Understanding local coastal governance is key for analyzing policy responses to coastal hazards and SLR. Hawaiʻi’s beaches and coastlines are held under the public trust as a natural and cultural resource to be protected and preserved by the State by managing coastal development and sustainably adapting to coastal hazards^40,41^. The State owns all land up to the shoreline, which is defined as the highest wash of the waves annually, “other than storm or seismic waves”^40^. Structures within the setback distance from the shoreline are considered nonconforming, meaning new construction and major repairs are no longer allowed in this area^42^. The setback for O‘ahu was recently increased to 18 m with additions based on historic rates of erosion^43^. New private shoreline hardening structures (e.g. seawalls) are illegal, unless clearly demonstrated to be in the public interest^40,42^. Under these policies and regulations, retreat of private property is inevitable along many stretches of Hawai‘i’s coastline as sea level rises and structures become threatened by the encroaching shoreline; however, additional action from state and local government is necessary for implementation and enforcement. Though state law is clear in regards to prioritizing beaches over private development, historical hardening of the shorelines has persisted and remains contested^44–46^.

**Figure 1:**
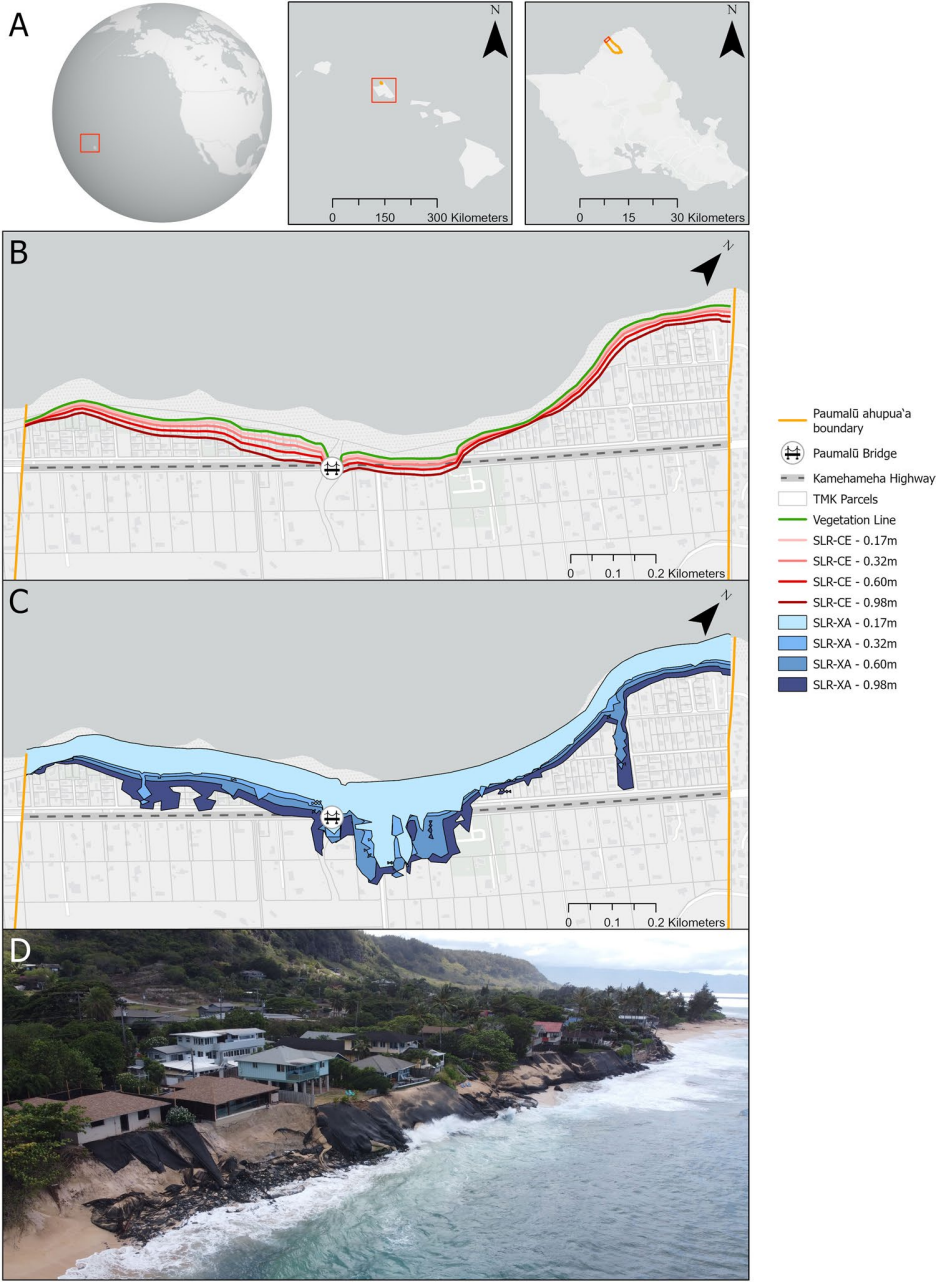
Study site of Paumalū ahupua‘a, located on the island of O‘ahu within the Hawaiian Islands (**A**). An aerial image displays a segment of the case study area demonstrating the chronic erosion and efforts to prevent further erosion with geotextile materials (photo credit: Renee Setter; panel **D**). A comprehensive visualization of datasets used in this study, including coastal erosion (SLR-CE; panel **B**) and exposure area (SLR-XA; panel **C**) projections at varying SLR levels in the case study area. The most recently available 2021 vegetation line (**B**) is included to illustrate the highest wash of the waves. Local infrastructure datasets include the highway (Kamehameha Highway) and the bridge over Paumalū Stream. TMK parcels are indicated in gray outlined boxes. Maps created in ArcGIS Pro^50^ and basemap provided by Esri^51^.

This paper quantifies the costs for retreat from the shoreline on the Hawaiian island of O‘ahu within the Paumalū *ahupua‘a* [a traditional Native Hawaiian socio-ecological subdivision of land (see Fig. 1)^47^]. The area is also known as Sunset Beach and is a popular resident and tourist destination for activities such as surfing. A significant portion of the existing public and private structures in the area are built on sand dunes, including a portion of Kamehameha Highway and a bridge^48^. In addition to transport infrastructure, the study area contains potable water and private stormwater management structures and pipes. Properties in this area are not connected to municipal wastewater, so they all have onsite sewage disposal systems (OSDS), consisting mainly of cesspools^49^.

The study site was chosen because it has experienced extreme erosion in recent years, with the maximum historical erosion rate in this area recorded at 0.32 m/year^52^. Erosion is exacerbated by SLR, which has resulted in multiple public and private structure failures in the study area, including separate incidents of the collapse of a home, a pool, and a bike path onto the beach and the relocation of lifeguard infrastructure^53–55^. Studies have shown that property owners are more likely to invest in defensive measures and later abandon dwellings at a risk threshold^56^. Autonomous coastal retreat has not occurred at this site and instead, many of the beachfront properties have geotextile materials (“sand burritos”) on the public beach to prevent further erosion of personal property (Fig. 1D), which were issued through temporary permits by the State in the past but are now illegal as the permits have all expired^57^. Beach renourishment is not a feasible long-term solution in this area due to the high-energy and dynamic conditions at this site^58^. Sand pushing is performed by the County to remediate erosion caused by foot traffic at Sunset Beach but the solution is temporary in the face of chronic erosion accelerated by SLR^59^.

Given that beaches are a part of Hawai‘i’s public trust doctrine and new private shoreline armoring is prohibited, the only long-term solution for adaptation to SLR-exacerbated chronic erosion in sandy beach areas like Sunset Beach is to retreat, with protection and accommodation being viable only as temporary solutions if the beach is to be maintained^58,60^. In addition, Hawai‘i’s moving shoreline implies that retreat of coastal development is inevitable under SLR.

## Retreat framework

**Figure 2:**
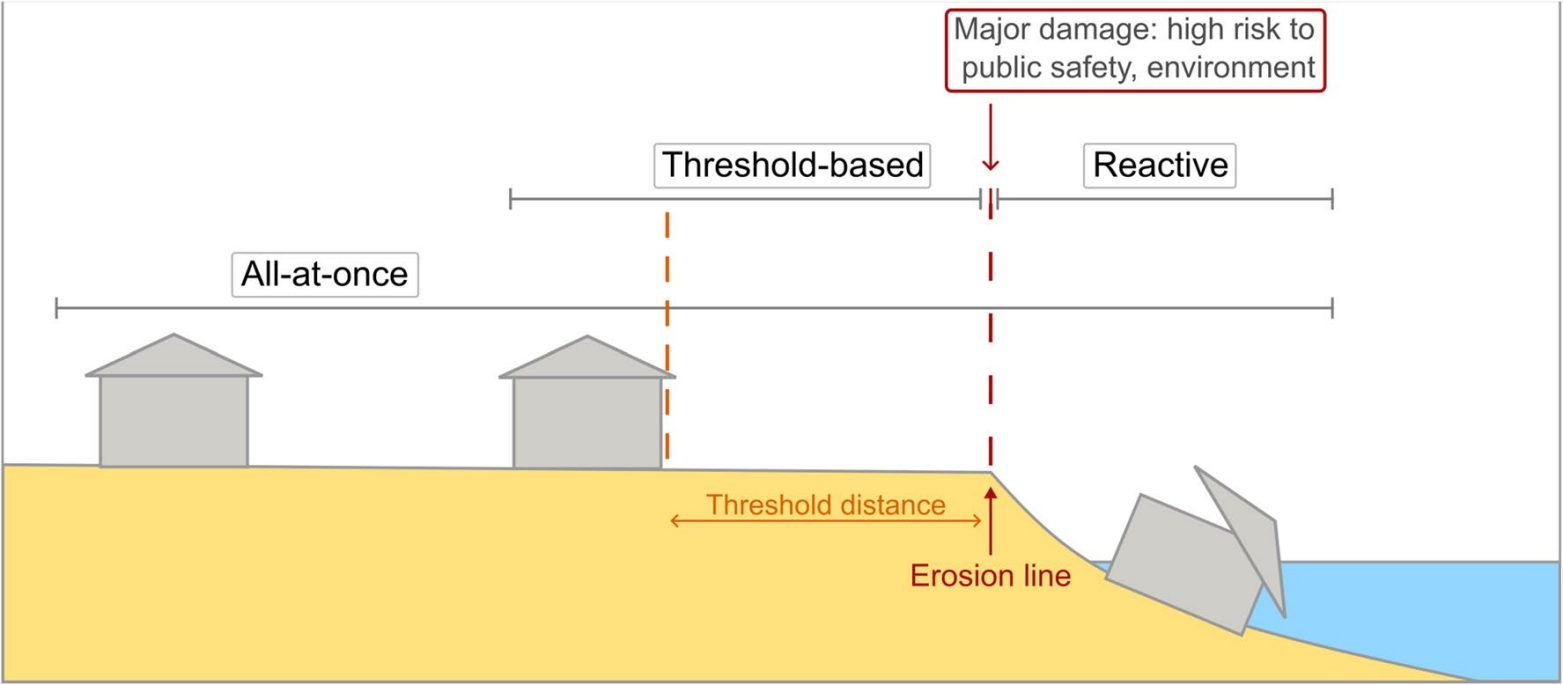
Depiction of retreat framework for the three retreat approaches: all-at-once, threshold-based, and reactive. Figure created in Inkscape^64^.

Retreat that is coordinated and done purposefully is called “managed retreat”^61^. The converse, retreat that is “unmanaged,” is a process that happens haphazardly and/or without shared intention. Here, we define three retreat approaches: all-at-once, threshold-based, and reactive (Fig. 2; “Methods”), as has similarly been defined in the literature^62,63^. All-at-once retreat is the managed, planned, and proactive removal and potentially relocation of communities, buildings, and infrastructure inland. In this approach, all considered properties are acquired and retreated simultaneously and as soon as possible. Threshold-based retreat postpones managed retreat until a predetermined trigger is reached. Here, we base our threshold on a local ordinance, such that the threshold distance is six meters away from the coastal erosion line and we assume dwellings must retreat once the distance to the erosion line is less than the threshold (see “Methods”). Threshold-based retreat is triggered on a parcel-by-parcel basis. Both all-at-once and threshold-based retreat enable mitigation of public safety and environmental damage risks. A reactive approach would generally be considered an “unmanaged” approach to retreat; however, there could still be shared intention between government and landowners in this approach, as well as means to lessening public risk. Reactive retreat occurs after major damage or a disaster event, where the only remaining option is evacuation. In this retreat approach, high levels of public safety and environmental damage risks, for example through the introduction of asbestos and lead into the nearshore environment, are potentially incurred. We use the projected erosion line to determine when major damage, such as a dwelling falling onto the beach area, may occur.

We estimate the costs of the three retreat approaches within the study area of Paumalū ahupua‘a. Costs quantified in this study include those accrued by federal, state, and county government as well as private actors (i.e. current homeowners). These include property acquisition costs (i.e. voluntary buyouts or eminent domain), structure removal and relocation (both private structures and public infrastructure), loss of property tax revenues, and private property loss. We did not include costs for litigation and enforcement, ecosystem restoration, ecosystem services, as well as other social, cultural, and environmental costs—though these could all be areas for future research. We develop scenarios to explore variation in cost estimates based on the uncertainty in coastal governance and implementation relevant to the chosen retreat approach. Specifically, whether the State enforces its definition of the legal shoreline as the highest annual wash of the waves or remains with the status quo of enforcement based on the erosion line.

Costs were determined based on SLR projections, property value assessor’s data, and geospatial infrastructure data (see “Methods”). SLR projections were used to identify the properties or parcel areas that would be impacted under different levels of increased exposure. The SLR projections included modeled projections of passive flooding (SLR-PF), annual high wave flooding (SLR-AHWF), coastal erosion (SLR-CE), and a cumulative exposure area that represents the summed coverage of all aforementioned SLR projection types (SLR-XA)^52^. Each of these projections model threats as induced and exacerbated by SLR (see “Methods”). The effects of SLR-PF in this study site is negligible due to the high elevations of existing homes on sand dunes^65^. We use SLR-CE as a proxy for the shoreline under a conservative determination of land transfer from private to public, as enforcement of the legal shoreline has been inconsistent (Fig. 1B). SLR-XA was used for a more comprehensive calculation, as it is dominated by SLR-AHWF in our study area, which is a close representation of the legal shoreline in Hawai‘i under strong enforcement (Fig. 1C). High- and low-cost ranges under each retreat approach are calculated based on the inclusive SLR-XA definition of the shoreline (i.e. all-at-once-XA, threshold-based-wave, reactive-full-loss) and the conservative SLR-CE definition (i.e. all-at-once-CE, threshold-based-veg, reactive-veg; see “Methods” for additional definitions) and all costs were brought to net present value in $2021 (see “Methods”). We utilize SLR projections under a RCP8.5 business-as-usual scenario for several increments across the century: 0.17, 0.32, 0.60, 0.98 m representing years 2030, 2050, 2075, and 2100 respectively. Property values for 2021 from the City and County of Honolulu Assessor’s office were used as a proxy for housing market value^66^. Geospatial datasets for parcels, dwellings, and infrastructure were used to determine impacted structures under varying SLR projections^49^. We do not account for the possibility of added dwelling density. We do not conduct a cost–benefit analysis but represent benefits of retreat by assessing the beach area gained over time under each retreat approach. Retreated parcel area becomes assumed beach area gained (see “Methods”).

## Results

**Figure 3:**
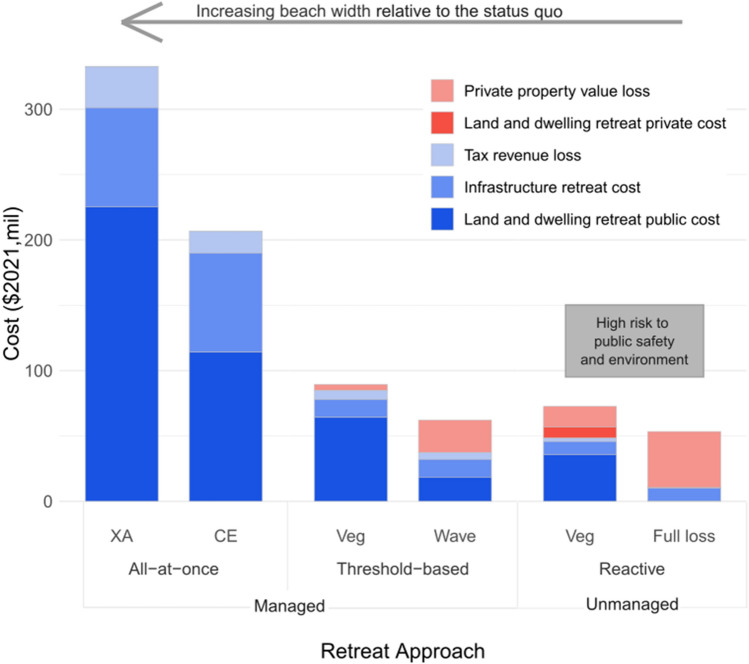
Costs of each retreat approach separated by type of cost and cost bearers. Costs borne to the public are indicated in various shades of blue and private costs are indicated in shades of red.

Our study finds the highest cost, overall and to the public, would occur under the all-at-once retreat approach, with the total cost between $207 and $333 million ($2021; Fig. 3). These costs are incurred mainly from the costs of acquiring properties at full market value. The all-at-once retreat approach is approximately four times more costly than threshold-based and reactive retreat. Threshold-based and reactive retreat have overlapping overall cost ranges due to the uncertainty of shoreline enforcement as the erosion line or the high wash of waves, with the cost for threshold-based retreat ranging between $62 and $89 million and reactive retreat between $53 and $73 million ($2021). The costs of threshold-based and reactive retreats are much lower than the all-at-once approach as costs of retreat are delayed, and allows more land to transfer from private to public ownership as the shoreline rolls inland with SLR. The low-end cost for the reactive retreat (reactive-full-loss scenario) is the lowest overall cost option as there are no acquisition costs since we assume the land no longer has any development value and the dwelling is substantially damaged. However, this scenario poses the highest risk to public safety and potential for environmental damages.

The distribution of costs to public versus private actors is greatly dependent on the retreat approach. Reactive retreat yields the lowest public financial costs ranging from $10 to $48 million ($2021; Supplementary Table S1). However, our reactive retreat cost estimates would require heavy enforcement from the state to ensure private actors pay for clean-up, and it does not account for the costs associated with high public safety risk and potential environmental damage, environmental contamination hazards, or temporary lack of beach access. Reactive retreat is the most costly option for private actors (ranging from $24 to $43 million), as they would lose significant, if not all property value and hold responsibility for structure clean-up due to public nuisance laws, though this would need to be backed up with enforcement. Threshold-based retreat, most likely prompted through condemnation, is the next best option for private actors, with estimated private property losses ranging between $5 and $25 million ($2021). The threshold-based approach is also a moderate option for public cost, ranging between $37 and $85 million ($2021). Additionally, there would be less safety risk and potential environmental damage in the threshold-based retreat approach compared to the similarly-priced reactive retreat. All-at-once retreat poses the largest public cost, as the entirety of costs are borne to the public through land and dwelling acquisition costs, infrastructure retreat costs, and tax revenue loss. Private landowners are financially best off under all-at-once retreat, most likely occurring through a voluntary buyout program, as no direct costs are incurred to the private actor. The loss of property tax revenue per retreat approach is much less than other costs in all retreat approaches, ranging between $0 and $32 million ($2021).

**Figure 4:**
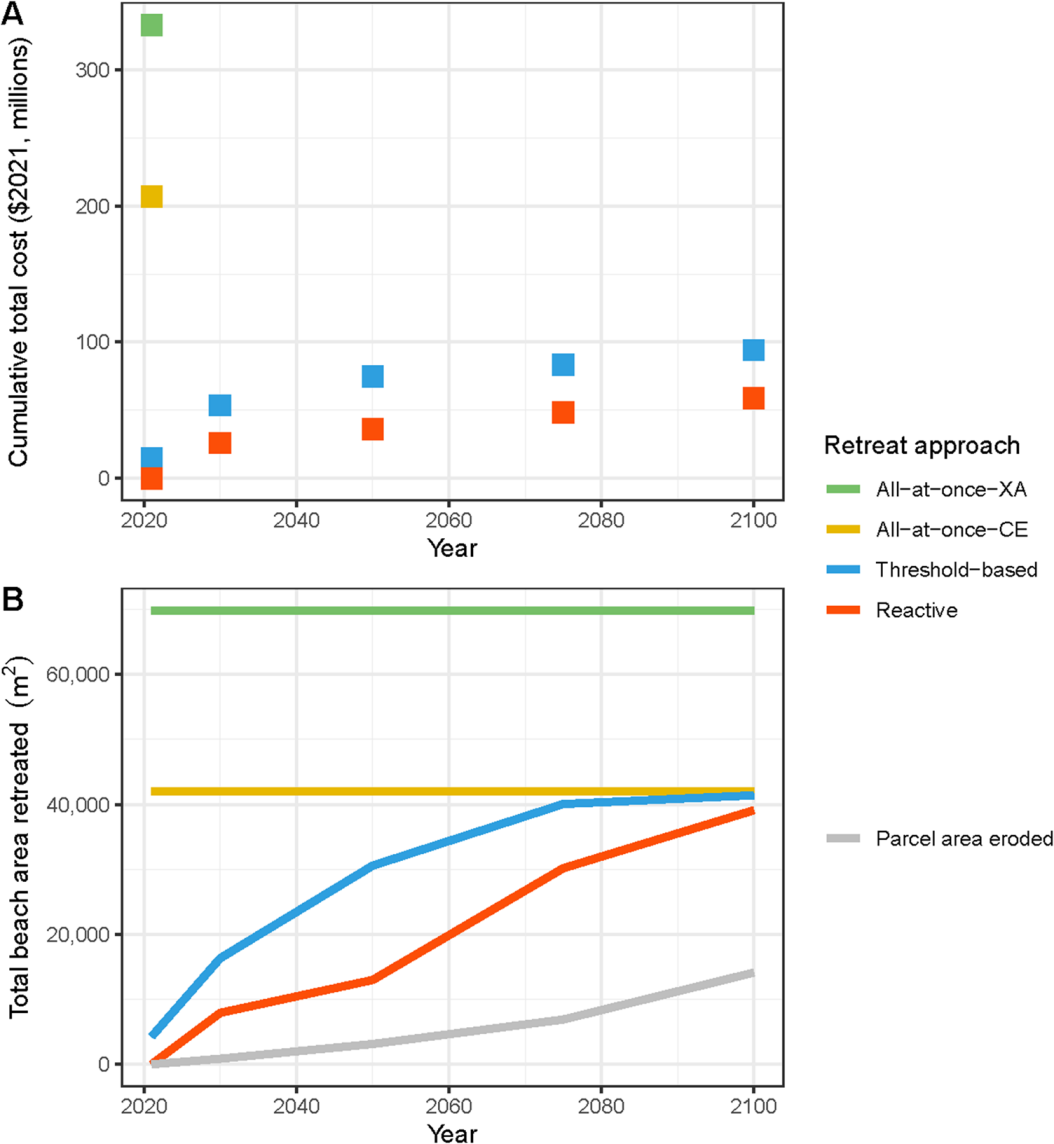
The average cumulative discounted total cost in $2021-millions for each retreat approach (**A**) and beach area gained (**B**) under each retreat approach are displayed over time. Both all-at-once retreat approaches (all-at-once-XA and all-at-once-CE) are highlighted here due to their wide ranges, whereas ranges for threshold-based and reactive retreat approaches are much smaller and similar. Parcel area eroded indicates sand dune land that is lost under SLR-CE.

Each retreat approach uses a different method to determine the number of parcels that retreat over time, influencing the cost distribution and beach area that could be gained within our study period. Under the all-at-once approach, all 52–88 parcels retreat immediately, with the range in number of retreated properties due to the variability of the definition of shoreline that is used (see “Methods” and Supplementary Table S2). Under the threshold-based approach, the number of parcels to retreat peaks in 2030 and fewer parcels retreat in increments throughout the century. Under the reactive approach, parcels would not begin to retreat until 2030 and continue at a similar rate of retreat through the end of the century. Due to the timing of parcel retreat, the all-at-once approach has a very high upfront cost. In comparison, threshold-based and reactive approaches have lower costs since more land transfers from private to public over time, which decreases the initial and incremental cost burden to the public for acquisition compared to all-at-once retreat, while also allowing for residents to retain their properties for longer (Fig. 4A). The area retreated over time is an important consideration for public safety and environmental protection. The all-at-once approach allows for greater beach area for a longer amount of time as we assume beach area to be gained when dwellings are immediately retreated, because nearly all properties in the study area are built on top of marine sand dunes (Fig. 4B). The potential beach area that could be immediately gained under the all-at-once approach is between 42,000 and 70,000 m^2^ (25 m and 42 m of average beach width, respectively). The threshold-based retreat approach gives the highest beach area gained per dollar and yields a greater amount of beach area added than the reactive approach. The threshold-based-wave approach is the greatest value with half the cost per beach area gained compared to the all-at-once and reactive approaches. The reactive approach has a significantly less amount of beach area restored throughout the century as most dwellings retreat closer to the end of the century. While it appears that total beach area gained converges for all retreat approaches at 2100 (Fig. 4B), the trends for beach area gained in each retreat approach would remain separate upon application of future SLR projections developed for the next century.

## Discussion and conclusion

Managed retreat led by local governments is only likely to be successful if existing, strong coastal policy and protection laws are in place to address SLR and enable retreat, as has been seen in New Zealand, Alaska, and California^67–69^. Managed retreat has the potential to transform norms surrounding climate change adaptation by prioritizing justice issues and considering climate risks rather than just facilitating the movement of people away from hazards, as the burden of adaptation should be equitably distributed^70–72^. For coastal communities considering managed retreat, the financial and non-financial costs are relevant to the type of approach. Effective adaptation requires solving for multiple objectives, including long-term cost minimization and implementation that minimizes harm^63,70,73^. Our Paumalū, Hawaiʻi example has a six-fold cost difference between the most expensive all-at-once-XA and least expensive reactive-full-loss retreat scenarios. Importantly, the area of our study site is just a 1.7 km segment of the 365 km total coastline of O‘ahu (Fig. 1A), and much of the coastline faces a similar need for SLR adaptation response and funds to do so. Thus, the most realistic way forward for communities with limited public funds may be through trigger-based approaches such as threshold-based and reactive retreat, although it is important to consider the additional costs (clean-up of structural debris, public safety risks, and contaminant pollution) incurred during reactive retreat that are largely mitigated by threshold-based retreat. Comparatively, threshold-based and reactive retreat cost around the same with a maximum difference of 1.5-fold, which are driven mainly by differences in assumptions about debris removal.

Prioritizing public safety should be at the forefront of SLR response planning, as previous case studies have displayed risky disorganization and ineffective management for relocation in an unmanaged retreat^72^. Retreating development reactively entails collapsed structures onto the beach slope that are exposed to the waves, increasing the risk of exposure to hazardous pollutants and beach conditions for those residing in and using the nearby areas. While reactive retreat is the most financially affordable approach in our study, it is of a similar cost to threshold-based retreat; policymakers may find threshold-based retreat worth the marginally greater cost due to its added benefits of greater beach area and public and environmental safety. The threshold-based approach has potential for preserved public safety and environmental protection because it maintains a larger area for beach migration over a longer period of time, allowing for increased beach width and less potential for damage and risk from structures falling into public beach area (Fig. 4B). Moreover, it has been found that other beaches on O‘ahu gain value with added beach width and it is possible that there is also greater public value for increasing beach width within our study area^74^. The threshold-based approach is additionally priced at a much affordable level than the all-at-once approach, with costs spread between both public and private actors as well as spread out over a longer time period than the single cost payment of the all-at-once scenario.

For threshold-based retreat, a predetermined threshold defines when coastal development needs to be retreated. All stakeholders must work together to agree upon the level of risk that is acceptable and the respective threshold level that once triggered, enables retreat^62^. Coastal communities should collaboratively determine a retreat policy that protects homeowners and public safety but does not retreat prematurely^75^. Since community inclusion is imperative to successfully execute threshold-based retreat, this approach may help to reduce societal resistance to retreat^76^. Additionally, post-retreat plans should be crafted to determine the use and jurisdiction of the retreated area, possibly through dune restoration or land trusts^77,78^. For this study, a fixed distance of 6 m from the coastal erosion line was chosen because Hawaiʻi Administrative Rules § 13-5-2 uses this metric to determine when a structure is “imminently threatened” and has a seamless application to publicly available data. However, a dynamic threshold may be more sensible for certain coastlines with a history of erosion, where this threshold would be based on historical shoreline change rates^79^. Dynamic threshold-based retreat could be a more preferable pathway as it operationalizes retreat into manageable steps and implements a flexible, place-based approach where threshold distance depends on localized erosion or flood patterns under SLR. The geospatial data to study and implement a dynamic threshold was not available for our study site, thus collaboration with local coastal hazard modelers would be necessary to conduct this approach. Future work should incorporate an analysis of different threshold types and extents as well as benefits from retreat options in our case study area and beyond.

We recognize there are important limitations to our analysis. The assessor’s dataset used here reports land and structure values with a year-lag from market values and does not capture changing market dynamics. Infrastructure costs are also likely underestimated, because we consider only the study area affected, not the adjacent infrastructure that would also be impacted and required to realign. There is inherent uncertainty in the modeled SLR projection dataset, which could lead to slight inaccuracies in the timing and cost of each retreat approach. Furthermore, the SLR projection dataset extends only to 2100. However, the sea level would continue to rise beyond that date. To provide continuous cost and beach gain estimates, this method requires re-evaluation of additional properties to retreat in accordance with updated SLR-XA/CE models beyond 2100. This study focuses on financial costs of retreat and beach area changes but it does not fully capture the social, cultural, and environmental values nor represent the potential safety risks or environmental damages related to pollution remediation. Additionally, this study only focuses on sandy beach areas with residential homes, which keeps the further application of this method limited to similarly zoned areas. Future iterations of our methodology aim to include different development typologies (e.g. urban areas with sandy beaches, urban areas with waterfront, areas with sewer infrastructure), other SLR-induced coastal hazards (e.g. groundwater intrusion), and additional SLR responses as appropriate.

## Methods

In this study, we calculate the costs for three retreat approaches at five time increments. Projected SLR levels were used to determine the overlap onto parcel properties and infrastructure, then associated costs for retreat were calculated under each retreat approach at all temporal increments. The SLR projections represent the upper limit (83rd percentile) of the likely range from the RCP8.5 scenario of the Intergovernmental Panel on Climate Change 5th Assessment Report (CMIP5), though this is now considered an intermediate likelihood scenario^10,52^. The SLR projections model passive flooding (SLR-PF), annual high wave flooding (SLR-AHWF), coastal erosion (SLR-CE), and a cumulative exposure area (SLR-XA)^52^. This dataset was developed to model Hawai‘i-specific SLR hazards as a resource for local planners and policymakers^30^. It models coastal processes relevant in this region including waves, groundwater inundation, and historic shoreline erosion change rates to quantify a comprehensive projected land area exposure to multiple SLR hazards^52^. These projections were used at multiple temporal increments at years 2030, 2050, 2075, and 2100 representing 0.17, 0.32, 0.60, and 0.98 m of SLR respectively as available in the original projection dataset. Storm surges are rare in Hawai‘i and therefore are not modeled in this dataset. The relative location of SLR projection levels was then compared with assets including parcels, dwellings, and infrastructure^49,66^. Potable water infrastructure data is unavailable for security reasons, but we assume that the infrastructure is embedded within roads. The overlap of SLR projection lines on geospatial asset locations informed the identification of impacted structures under SLR and the associated costs for each retreat approach at each timestamp (see Supplementary Table S3 for unit costs). Tax revenue loss assumes that taxes decrease proportionally to the value of a parcel after applying the land transfer and retreat timing methodologies. We calculate future potential taxes based on 2022 Residential and Residential A tax rates for O‘ahu^80^. Private property value loss is the difference between 2021 assessed values and values remaining after land transfer and/or loss in dwelling value.

Costs for retreat were calculated for three different retreat approaches with a range of estimates including both a conservative cost based on the enforced shoreline and a higher cost based on the legal shoreline. The criteria for each retreat approach are summarized in Table 1.

**Table 1:** Summary of retreat approach methods and criteria used to determine infrastructure and dwelling retreat.

Approach	Scenario	Dwelling retreat criteria	Land transfer criteria	Infrastructure retreat criteria
All-at-once	All-at-once-XA	0.98 m SLR-XA	Full value	0.98 m SLR-CE
	All-at-once-CE	0.98 m SLR-CE		
Threshold-based	Threshold-based-veg	≤ 6 m from SLR-CE	SLR-CE	Harden ≤ 6 m SLR-CE, retreat all at 2100 using 0.98 m SLR-CE
	Threshold-based-wave		SLR-XA	
Reactive	Reactive-veg	Intersecting or seaward of SLR-CE	SLR-CE	Harden road intersecting or seaward of SLR-CE, retreat all at 2100 using 0.98 m SLR-CE
	Reactive-full-loss		No value left	

Under the managed, all-at-once retreat approach, all existing coastal structures are assumed to be acquired at full market value rates ($2021 assessed values). After acquisition, private structures such as houses and on site sewage disposal systems (OSDS) are demolished and removed and public infrastructure such as roads, bridges, and water infrastructure projected to be affected by 2100 are removed and relocated inland immediately, all at expense to the public. The infrastructure cost includes the removal cost, eminent domain cost for private properties where the new road would need to be realigned, and rebuilding cost. Local neighborhood roads would only be removed, as the properties using these roads are assumed to already be retreated. Under the all-at-once approach, all coastal development seaward of the projected 2100 SLR line would retreat immediately. The lower range cost for this approach, using the enforced shoreline, is the all-at-once-CE scenario and employs the 0.98 m SLR-CE projection line. The higher range cost for this approach, using the legal shoreline, is the all-at-once-XA scenario and employs the 0.98 SLR-XA projection line. The length of infrastructure to retreat is determined by the 0.98 m SLR-CE line for both all-at-once-XA and all-at-once-CE scenarios.

The managed, threshold-based retreat approach occurs once a predetermined threshold is triggered and utilizes an incremental approach to acquisition, likely through a voluntary buyout. Homeowners are compensated for their dwelling and the property landward of the SLR-XA or SLR-CE line, assuming there is a linear relationship between land loss under SLR threats and land parcel value. However, there are market responses to SLR threats that may be unaccounted for in this simplified linear devaluation of property^81,82^. Land acquisition costs, dwelling removal, and OSDS removal are at the expense of the public. Under the threshold-based approach, a parcel will retreat if the projected SLR line at the given time period is less than or equal to 6 m away from the seaward edge of the building footprint but has not yet intersected the building footprint. We use 6 m as the threshold since a property is considered “imminently threatened” and could potentially qualify for a short-term emergency hardening permit if an eroding shoreline is 6 m away from a dwelling^83^. If a parcel is located more than 6 m from the SLR line in one time period but intersects the SLR line in the next time period, we assume the threshold-based retreat would occur in the year halfway between the two time periods. If a parcel fulfills the criteria for threshold-based retreat at multiple time periods, it is assumed to retreat at the first occurrence. Acquisition costs are expressed in discounted present value in $2021 using a discount rate of 2.6%. This is a long-run discount rate for real estate, estimated in a recent paper^84^. Roads are hardened until the entire segment of infrastructure is within 6 m from the SLR-CE line and a single retreat and realignment occurs at 2100. The values are in $2021 using a 3% discount rate for public infrastructure, which is akin to the long-term bond rate. Higher rates of discount would further the gap of our estimates between scenarios. The lower range cost for this approach, using the enforced shoreline, is the threshold-based-veg scenario, which assumes the value of land transfers with the SLR-CE line and dwelling value is fully compensated at the time of acquisition. The higher range cost for this approach, using the legal shoreline, is the threshold-based-wave scenario, which assumes that the value of land transfers with the high wash of the waves and no dwelling value is compensated.

The unmanaged, reactive retreat approach occurs after a dwelling has likely collapsed or been considerably damaged, creating public safety and environmental risks, and incurring large clean-up costs. In our model, reactive retreat occurs when the projected SLR-CE line is intersecting or landward of the building footprint, representing when a dwelling has likely fallen onto the public beach. In this retreat approach, the dwelling value is zero and the clean-up cost is borne by the dwelling owner. Because houses cross the SLR line at different times throughout the study period, reactive retreat is a more piecemeal approach. Roads are hardened until the event of a single retreat (removal and realignment) when the infrastructure is critically affected and the SLR-CE line has intersected or is landward of the infrastructure. The lower range cost for this approach is the reactive-full-loss scenario where we assume no land value remains because the land has no development potential left as it violates county setback and development regulations. The higher range cost for this approach is the reactive-veg scenario where we assume the remaining land has value and is acquired at expense to the public and includes higher cleanup costs.

Beach gained under each retreat scenario is calculated by summing the area of parcels that would retreat at each timestamp under each retreat approach. Since most properties in this area are built on sand dunes, we assume that once the property retreats, the remaining area becomes part of the beach area. Erosion area is calculated by summing the parcel area that becomes seaward of the SLR-CE line for each timestamp.

## Supplementary Information

Supplementary Tables.

## Data Availability

The sources of all data used in this analysis are cited in the paper.
