# Supplementary material for: Managing retreat for sandy beach areas under sea level rise
Source: Sci Rep. 2023 Jul 24;13:11920. doi: 10.1038/s41598-023-38939-4 (PMC10366347; doi:10.1038/s41598-023-38939-4)
Supplement: Supplementary file 1 — Supplementary Tables. [file 41598_2023_38939_MOESM1_ESM.docx]

# Supplementary Information

**Supplementary Table 1.** The costs of each retreat approach presented in $2021, millions.

| **Cost type ($2021, mil)** | **Managed** | | | | **Unmanaged** | |
| --- | --- | --- | --- | --- | --- | --- |
|  | **All-at-once** | | **Threshold-based** | | **Reactive** | |
|  | **XA** | **CE** | **Veg** | **Wave** | **Veg** | **Full loss** |
| Land and dwelling retreat - public cost | $225.3 | $114.1 | $64.2 | $18.5 | $35.7 | - |
| Land and dwelling retreat - private cost | - | - | - | - | $8.5 | $0.4 |
| Infrastructure retreat cost | $75.9 | $75.9 | $13.6 | $13.6 | $10.1 | $10.1 |
| Tax revenue loss | $31.6 | $16.7 | $7.0 | $5.2 | $2.5 | - |
| Private property value loss | - | - | $4.6 | $24.8 | $15.8 | $42.9 |
| Total costs and losses | $332.9 | $206.7 | $89.4 | $62.1 | $72.5 | $53.4 |

**Supplementary Table 2.** The number of parcels and buildings that would retreat under each retreat approach over time.

|  | **Retreat approach** | **Now (2021)** | **By 2030** | **By 2050** | **By 2075** | **By 2100** | **Total** |
| --- | --- | --- | --- | --- | --- | --- | --- |
| **# of parcels** | All-at-once-XA | 83 | - | - | - | - | 83 |
|  | All-at-once-CE | 52 | - | - | - | - | 52 |
|  | Threshold-based | 8 | 17 | 12 | 12 | 3 | 52 |
|  | Reactive | - | 16 | 6 | 18 | 12 | 52 |
| **# of buildings** | All-at-once-XA | 138 | - | - | - | - | 138 |
|  | All-at-once-CE | 63 | - | - | - | - | 63 |
|  | Threshold-based | 8 | 20 | 15 | 15 | 5 | 63 |
|  | Reactive | - | 17 | 8 | 23 | 15 | 63 |

**Supplementary Table 3.** Per unit costs of retreat.

| **Cost Types** | **Cost/Unit** | **Scenarios** | **Source** |
| --- | --- | --- | --- |
| Property value | variable | All | (City and County of Honolulu Department of Budget and Fiscal Services, 2021) |
| Bridge retreat | $40,000,000 per mile; $94,000 per foot ($24,854,848 per km; $24,855 per meter) | All | Estimated based on existing Department of Transportation bridge replacement projects [(Department of Transportation Highways, 2019, 2021)](https://www.zotero.org/google-docs/?83xzpA). The total estimated cost is divided by the length of the bridge. |
| Bridge retrofitting | $20,000 per foot ($65,617 per meter) | All | Estimated based on existing bridge rehabilitation projects (such as Koukouai bridge) found in [County of Maui (2020)](https://www.zotero.org/google-docs/?9AFW7b). The total estimated cost is divided by the length of the bridge. |
| Road realignment | $70,000 per foot ($229,659 per meter) | All | [(Francis et al., 2019)](https://www.zotero.org/google-docs/?gYOa2b) |
| Single-lane road removal | $10 per foot ($33 per meter) | All | [Hometown Demolition (2022)](https://www.zotero.org/google-docs/?F61iSB) estimates $1-$3 per sq ft for asphalt removal. [National Association of City Transportation Officials (2013)](https://www.zotero.org/google-docs/?lzraOE) estimates the average width of a lane is 10 ft. Thus a conservative estimate for single-lane road removal is $10 per ft. |
| Eminent domain of landward properties for highway retreat | $386 per foot ($1,266 per meter) | All | For properties just mauka of the SLR-CE projection, the total parcel cost was divided by the total area (sq ft) and multiplied by 20 ft, per the [National Association of City Transportation Officials (2013)](https://www.zotero.org/google-docs/?XHIwdq) estimate for the average length of a two-laned highway. We assume only the width of the highway is subject to eminent domain. |
| New shoreline hardening | $10,000 per foot ($32,808 per meter) | All | [(Francis et al., 2019)](https://www.zotero.org/google-docs/?StP2td) |
| Decommissioning/ replacement of potable water mains | $1,443 per foot ($4,734 per meter) | All | Estimated based on [Teague (2017)](https://www.zotero.org/google-docs/?2nQfAo), which reported the annual cost to replace 1% of the 2,100 miles of water pipes on Oʻahu is ~$160 million. Conversion to cost per ft resulted in $1,443 per ft. Personal correspondence with a Board of Water Supply official confirmed this estimate. |
| Demolition of dwellings | $8,000 per average-sized home (~1,500 ft^2^ or ~139 m^2^) | All-at-once;  Threshold-  based | Estimated based on average values obtained from quote requests from local residential demolition companies. |
| Decommissioning of OSDS | $2,000 per cesspool | All-at-once;  Threshold-  based | Babcock et al. (2019) |
| Structural debris clean-up | Post and pier, dwelling stays intact: $10,000 to 25,000 per average-sized home (~1,500 ft^2^ or ~139 m^2^)  Slab foundation, dwelling breaks apart and falls into ocean: $100,000 to $200,000 per average-sized home (~1,500 ft^2^ or ~139 m^2^) | Reactive | Estimated based on average values obtained from quote requests from local engineering and construction companies. |
